# Supplementary material for: NIMO: A Natural Product-Inspired Molecular Generative Model Based on Conditional Transformer
Source: Molecules. 2024 Apr 19;29(8):1867. doi: 10.3390/molecules29081867 (PMC11053988; doi:10.3390/molecules29081867)
Supplement: Supplementary file 1 [file molecules-29-01867-s001.zip › molecules-2925617-supplementary.pdf]

Supplementary Materials

1. Motifs analysis

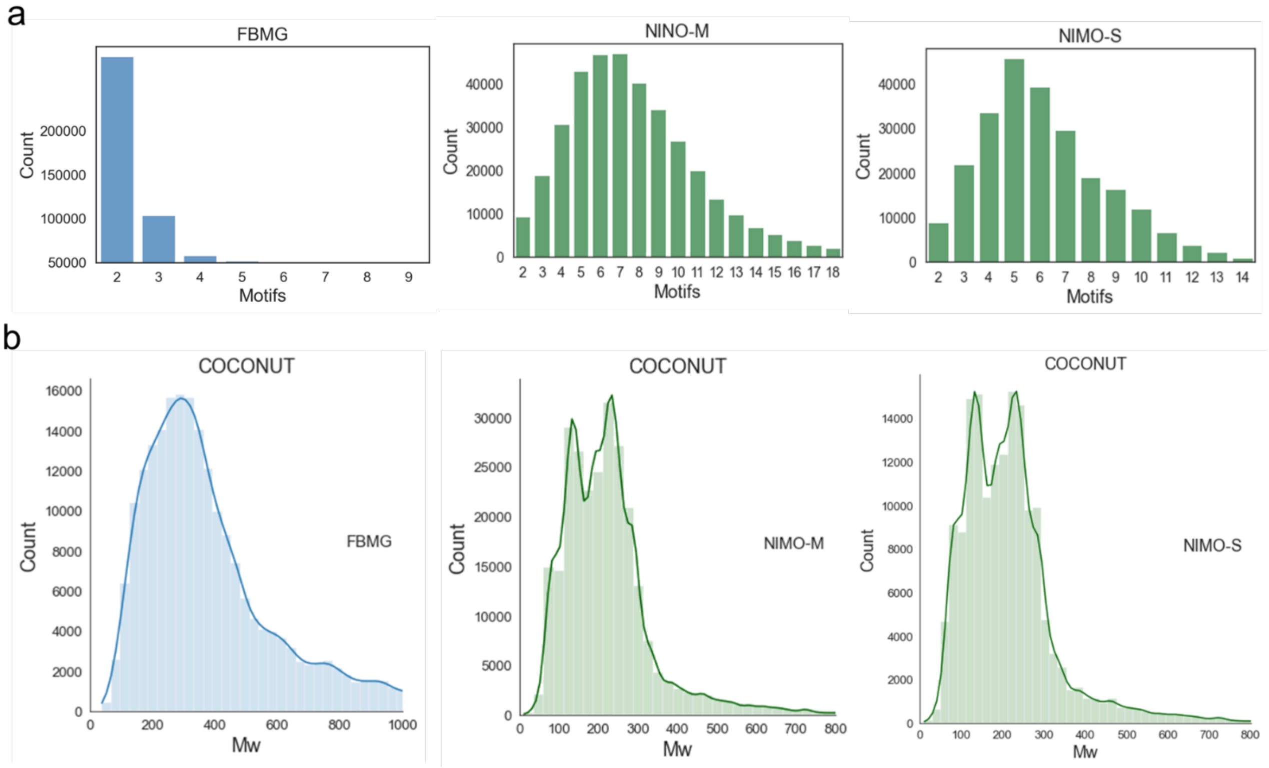

Figure S1. Overview of motifs generated by fragment-based models on the COCONUT dataset. (a) The distribution of the number of motifs existed in each motif sequence generated by three models FBMG, NIMO-M and NIMO-S, respectively. (b) Distribution of the weight of motifs from vocabulary built on three models FBMG, NIMO-M, and NIMO-S, respectively.

Figure S1 illustrated the comparison result of motif extraction among fragment-based models. **Figure S1a** shows that most natural products can only be decomposed into two motifs in FBMG, which was not conducive to adequate extraction of substructure features of natural products. In contrast, NIMO had a higher fragmentation efficiency and produced more motifs for each molecular structure from natural products. **Figure S1b** intuitively showed the differential distribution of motif weight from FBMG and NIMO, respectively. Compared to NIMO, there were heavier motifs in FBMG according to the median and mean values in **Table S1**. The common substructure of natural products cannot be fully extracted by FBMG, thus resulting in the greatest motif size, quantitatively.

Table S1. Statistics of motif size and motif weight from three models FBMG, NIMO-M and NIMO-S.

Models	Motif size	Median of motif weight (g/mol)	Mean of motif weight (g/mol)
FBMG	213,447	335.4	407.2
NIMO-M	161,967	200.3	218.5
NIMO-S	75,382	224.3	238.2

In the generation task, the validity of the 5,000 molecules generated by NIMO-M-O dropped down to 75.12%, while NIMO-M achieved **99.3%.** To investigate how the different effects of motif info improved the performance of the proposed model, we conducted the ablation study on the following variants of NIMO.

- NIMO-M-O was the variant of NIMO-M that removed the motif info of the input sequence.
- NIMO-S-O was the variant of NIMO-S that removed the motif info of the input sequence.

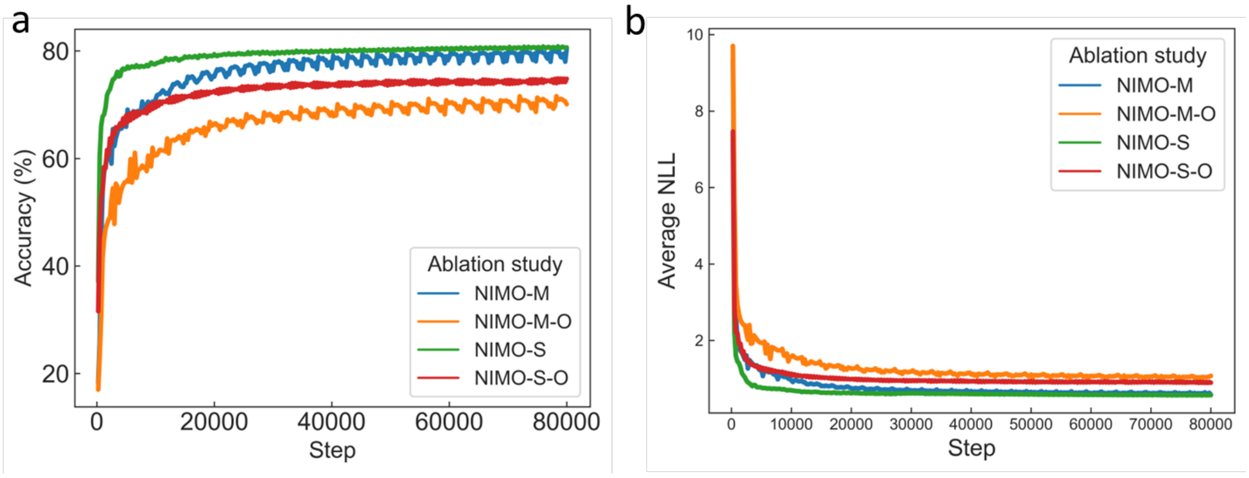

Figure S2. (a) The accuracy curves of different variants of NIMO during model training. (b) The average negative log-likelihood (NLL) curves of different variants of NIMO during model training.

The ablation experiments results were shown in Figure S2. We overall observed that NIMO improved the accuracy of model training with reduced average NLL and faster convergence in comparison to NIMO-M-O and NIMO-S-O. It indicates that the motif info of the input sequence contributed to help the model to jointly capture rich semantic correlation features extracted from

origin motif sequences.

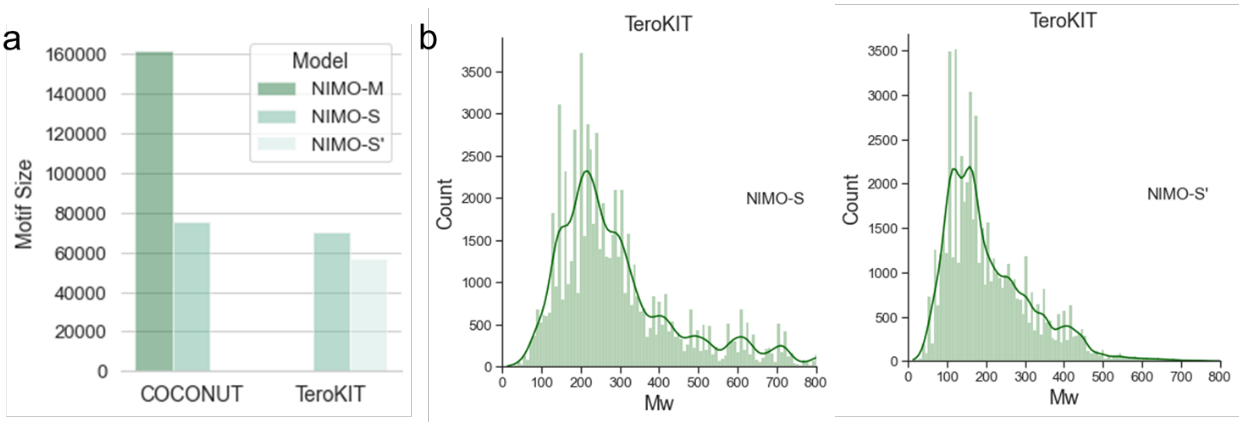

Figure S3. Overview of motifs generated by NIMO. (a) Histogram of motif size from vocabulary built on three models, NIMO-M, NIMO-S and NIMO-S'. (b) Distribution of the weight of motifs from vocabulary built on NIMO-S and NIMO-S', respectively.

2. Benchmark

To quantitatively assess the performance of the proposed NIMO as well as other published models in the field of molecular generation, we run through relevant and compatible metrics from the two major benchmarking suites including generic conditional metrics and the well-established MOSES[1].

2.1. Conditional metrics

The validity refers to the percentage of the generated chemically valid molecules. The rest conditional evaluation metrics are calculated by the equation:

$$Uniqueness = \frac{\text{nonduplicate and valid molecules}}{\text{valid molecules}} \tag{1}$$

$$Novelty = \frac{\text{not in train set unique molecular}}{\text{unique molecules}} \tag{2}$$

2.2. MOSES metrics

We downloaded the MOSES test code from their official repository https://github.com/molecularsets/moses. The well-established MOSES evaluation metrics are the main benchmark in the field of de novo molecular generation. In the MOSES metrics calculation, the baseline data are provided by the COCONUT[2] dataset and processed on demand. This means

that the default training data and test data are replaced with the corresponding data from COCONUT. The following summarizes the descriptions of MOSES evaluation metrics in this work.

Frechet ChemNet distance (FCD): Frechet ChemNet distance is calculated using activations of the penultimate layer of a deep neural network ChemNet trained to predict biological activities of drugs. Here, using training set (R) and the generated molecules (G). In short, FCD is defined as follows:

$$FCD(G,R) = \| \mu_G - \mu_R \|^2 + T_r(\sum G + \sum R - 2(\sum G \sum R)^{1/2}) \quad (3)$$

where μ_G, μ_R are mean vectors and ΣG, ΣR are full covariance matrices of activations for molecules from sets G and R respectively.

Nearest neighbor similarity (SNN): SNN refers to the average TaNIMOto similarity between the generated molecules (G) and the nearest neighbor molecules (T) in the test set.

$$SNN(G,T) = \frac{1}{|G|} maxT(m_G, m_T) \quad (4)$$

Fragment similarity (Frag): using cosine similarity to measure how frequently various molecular fragments appear in the generated set (G) and the test set (T). The calculation method of fragment frequency also obtains the BRICS function in RDKit[3], just like our NIMO model.

$$Frag(G,T) = cos(f_G, f_T) \quad (5)$$

Scaffold similarity (Scaf): using cosine similarity to compare the distribution of molecular fragments appear in the generated set (G) and the test set (T), The calculation method of fragment frequency obtains the extracting Murcko scaffolds in RDKit.

$$Scaf(G,T) = cos(f_G, f_T) \quad (6)$$

Internal diversity (IntDiv): the measure of the diversity for the generated set (G) by calculating the average taNIMOto coefficient (TC):

$$IntDiv(G) = 1 - \frac{1}{|(G)|^2} \sum_{(a,b)\in G} TC(m_a, m_b) \quad (7)$$

Novelty: the proportion of generated molecules (G) that are not in the training set (R).

$$Novel(R) = 1 - \frac{|G \cap R)|}{G} \quad (8)$$

Synthetic accessibility score (SAS): the metric is based on a combination of molecule complexity and fragment contributions.

2.3. Metrics for terpenoids generation

The success rate refers to the percentage of the molecules predicted as terpenoids by NPClassifier. The metrics related to the ring systems are calculated by the equation:

$$Coverage = \frac{\text{unique rings from generated set and exist in the training set}}{\text{the number of ring systems from generated set}} \quad (9)$$

$$Recovery = \frac{\text{unique rings from generated set and exist in the training set}}{\text{the number of unique rings from generated set}} \quad (10)$$

The metrics related to the functional groups are calculated by the equation:

$$Coverage = \frac{\text{unique functional groups from generated set and exist in the training set}}{\text{the number of functional groups from generated set}} \quad (11)$$

$$Recovery = \frac{\text{unique functional groups from generated set existed in the training set}}{\text{the number of unique functional groups from generated set}} \quad (12)$$

3. Scaffold-based scenario

We designed a scaffold-based scenario to evaluate the performance of NIMO-S. First, two scaffolds were randomly selected from the terpenoid training set and are shown in the left center of Figure S4. The scaffolds were surrounded by some structures obtained by sub-structure search towards training dataset. Second, we searched for the motifs corresponding to the two scaffolds by retrieving the vocabulary constructed by the NIMO-S. The motifs as core scaffolds were shown in the right center of Figure S4. The compounds surrounding the motifs were generated by the NIMO-S.

As shown in Figure S4a, the compounds T4-5 are modified at the undefined extension site corresponding to Motif 1. Thus, some similar structures to the partial training set cannot be generated by NIMO-S due to the restricted extension sites like C-8. Besides, the compounds T6-7 exhibit derivation into a hexameric ring at two adjacent derivation sites (C-3, C-4), and the NIMO-S has not yet implemented such a functional operation. Therefore, rough substructure matching leads to unfair comparisons between the training set and the generated set. It is suggested to clarify the desired scaffold-derived sites or try to define different scaffold extension sites to obtain more diverse structures. But otherwise, the structures generated by NIMO-S are similar to that of the training set. For example, compound S5 showed a similarity of 0.92 to compound T1 based on RDKit fingerprint algorithms. Compounds generated by NIMO-S reproduce the same functional groups as that in the training set such as ester groups, hydroxyl groups and carboxylic acids. There are also the same functional group modifications at the same extension sites like methyl group/carboxylic acids at C-6 of Scaffold 1 (gray circles). Meanwhile, NIMO-S decorates different substructures into attachment points to generate diverse derivatives such as a long side chain at C-10 of Scaffold 1 (red circles).

As shown in Figure S4b, the second type of polycyclic scaffold is common in triterpenoids. NIMO-S can reproduce the same modifications at conserved sites to produce similar molecular structures. For example, bis(methyl) at C-20 of Scaffold 2 (purple) is retained as well as isopropenyl group at C-14 (red). In addition, NIMO-S allows modifications with different functional groups, e.g., novel trimethylamine at c-14 (green circles) and long side chain at C-16 (bule circles).

Figure S5 illustrated the chemical space of the data sets defined by molecular fingerprints. As we can see, generated molecules related to scaffold 1 do not completely cover the training space due to the above-mentioned restricted extension sites. In contrast, generated molecules related to scaffold 2 displayed a higher coverage rate. Furthermore, NIMO-S can produce novel molecules outside the training set (arrows in Figure S5).

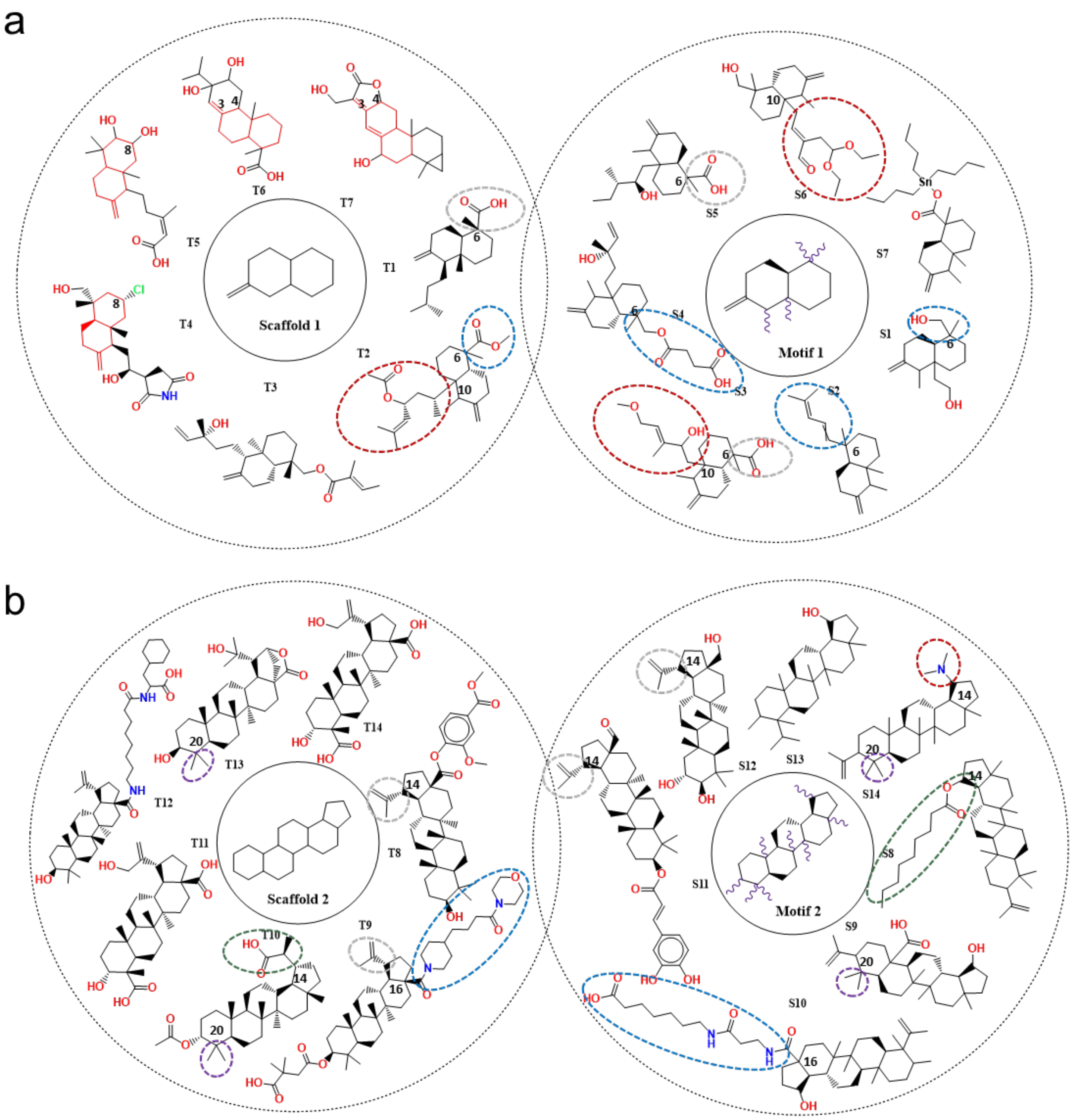

Figure S4. The additional cases of derivatives generated by NIMO-S in a scaffold-based scenario. Two motifs corresponding to the randomly selected scaffolds were used for molecular generation as core seeds, as shown in the center. The scaffolds are surrounded by compounds from the terpenoid training set, while the motifs are surrounded by compounds generated by NIMO-S.

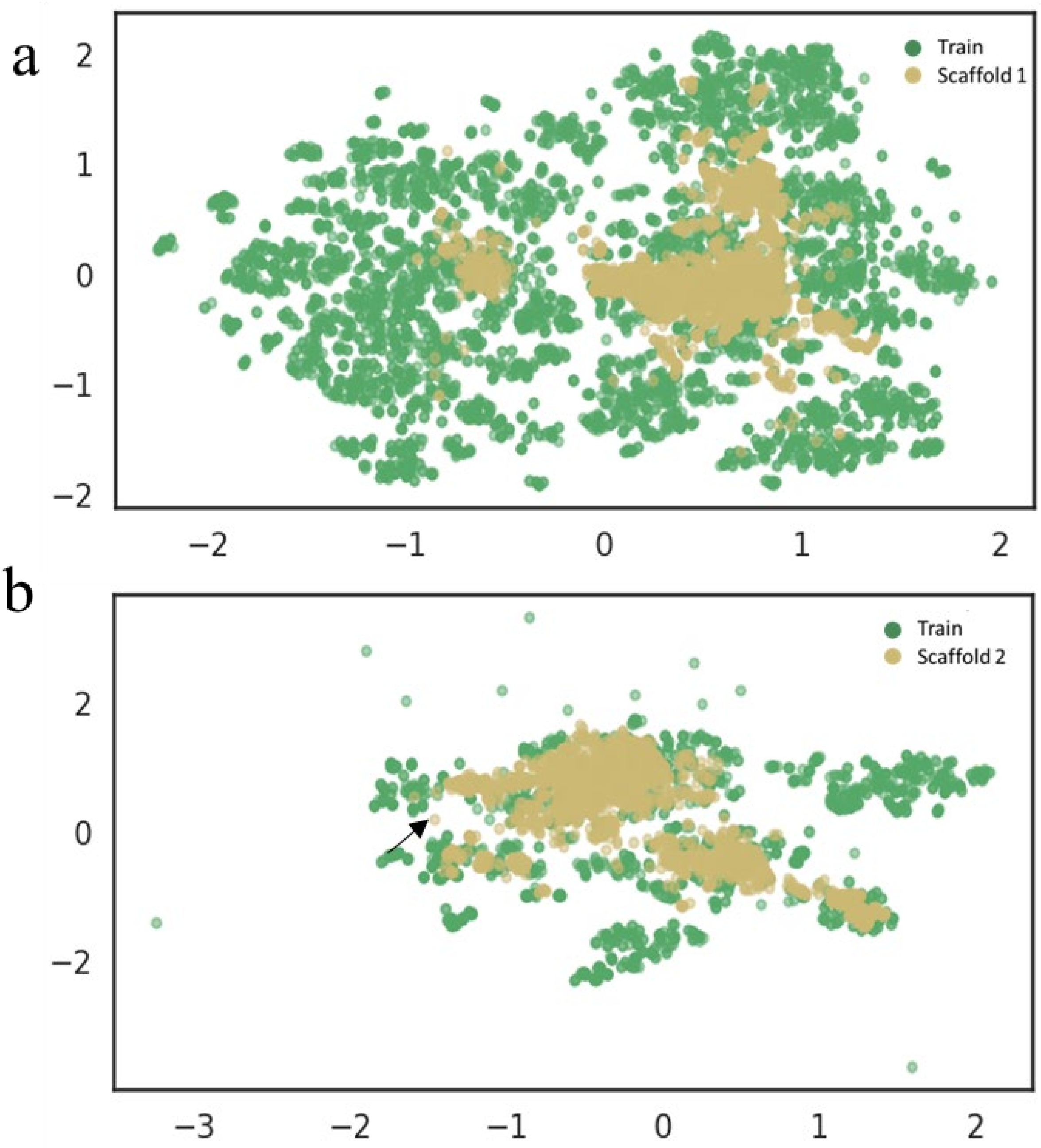

Figure S5. The t-SNE plots of the compounds corresponding to scaffolds. Green circle stands for compound in the training set. Yellow circle stands for compound generated by NIMO-S. By statistics, there are 6,250 compounds containing the structure of scaffold 1, and 1,834 compounds containing the structure of scaffold 2 in the training dataset. Herein, 5,000 compounds are generated by NIMO-S.

4. Antimalarial activity prediction

First, a full-scale prediction of antimalarial activity for compounds in the COCONUT dataset was performed on MAIP[4] (Malaria Inhibitor Prediction). A model score returned by the platform. The higher the score is the more likely the compound is predicted to be active. The enrichment factor (EF) is the hit rate (the proportion of active compounds) within a defined sorted fraction divided by the total hit rate. It is defined by:

$$EF[X] = \frac{P[X]}{N[X]} \cdot \frac{N}{P} \tag{13}$$

where X is the user-defined fraction, P[X] the number of actives in the fraction, N[X] the total number of compounds in the fraction, P the total number of actives and N the total number of compounds.

In this work, MAPI web-service automatically calculated this metric at 1 % ,10 % and 50%, respectively. The three vertical dashed lines in Figure S6a, from left to right, reveal that top 50% contains 372,493 compounds with the highest scores, which are all over 20.07; top 10% contains 74,498 compounds with the highest scores, which are all over 44.36; top 1% contains 7,449 compounds with the highest scores, which are all over 80.4. Because there is no normalized score, the user defines 44.36 as a selection threshold to label the train set. The model scores of antimalarial activity prediction on MAIP for 5,000 valid molecules generated by NIMO-M and MCMG[5] are showed in Figure S6b and Figure S6c, respectively. The result of model scores predicted on MAIP for 1,000 valid molecules generated by NIMO-M' are depicted in Figure S6d.

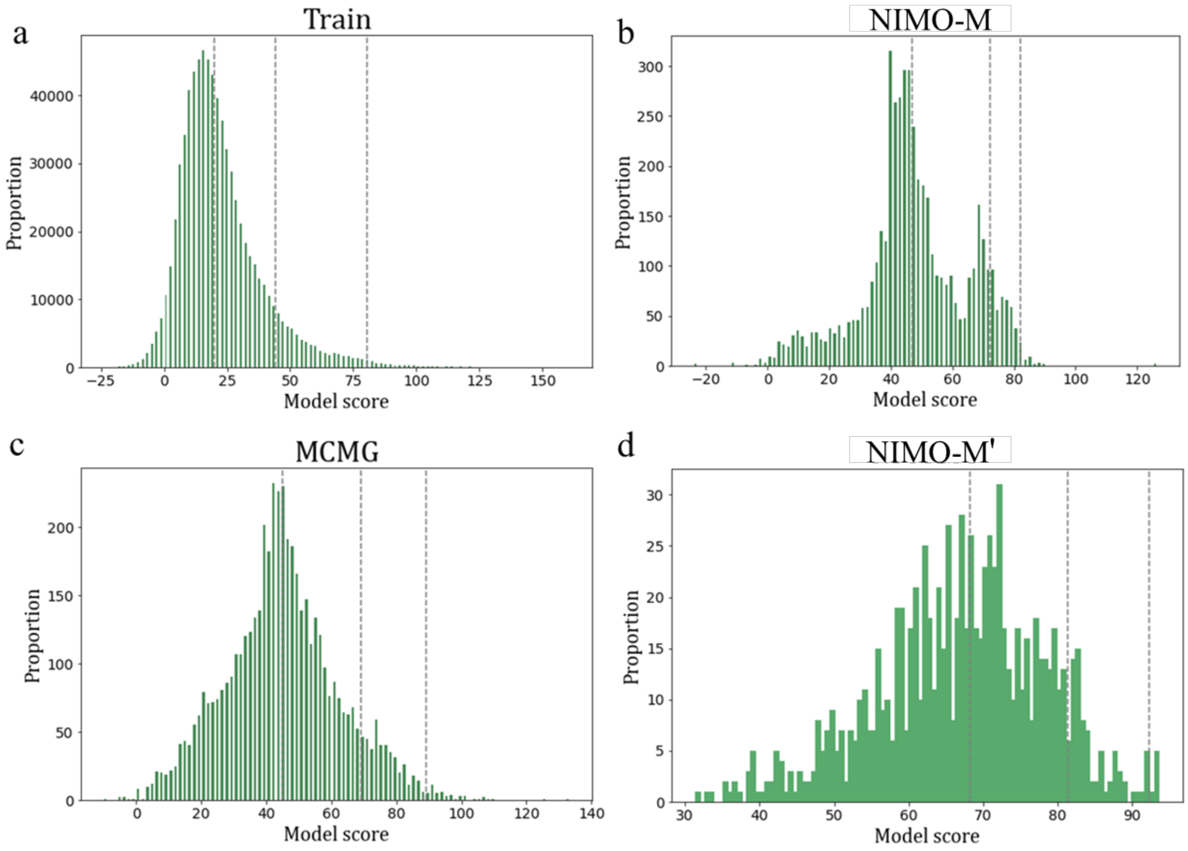

Figure S6. Distribution of antimalarial activity prediction scores on MAIP for four datasets. We hypothesized uneven contribution of fragments to predicted scores, e.g., dominant fragments sensitize the molecule to activity.

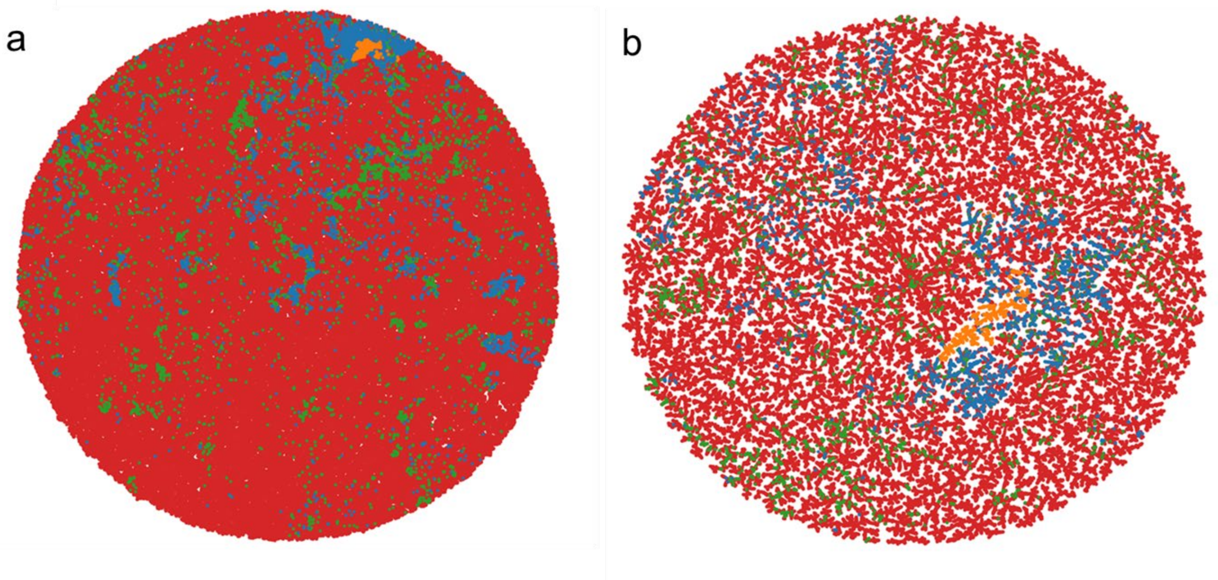

Figure S7. TMAP visualizations of chemical space distribution for four datasets. The compounds were indicated by red dots (train set), green dots (MCMG), blue dots (NIMO-M) and orange dots (NIMO-M’). (a) All of the compounds were taken into account. (b) Only compounds predicted to be ‘active’ were taken into account.

5. Design antibacterial discovery libraries

This task focuses on utilizing experimentally antibacterial active small molecules to build an integrated and systematic discovery pipeline, which allows in the identification of antibacterial species for new molecules generated by NIMO. In brief, we built an anti-bacterial dataset (255,788). A kNN classifier assists in further predicting targeted bacterium for the molecules generated by NIMO and scouting of candidates. The downstream task was conducted according to the workflow shown in Figure S8.

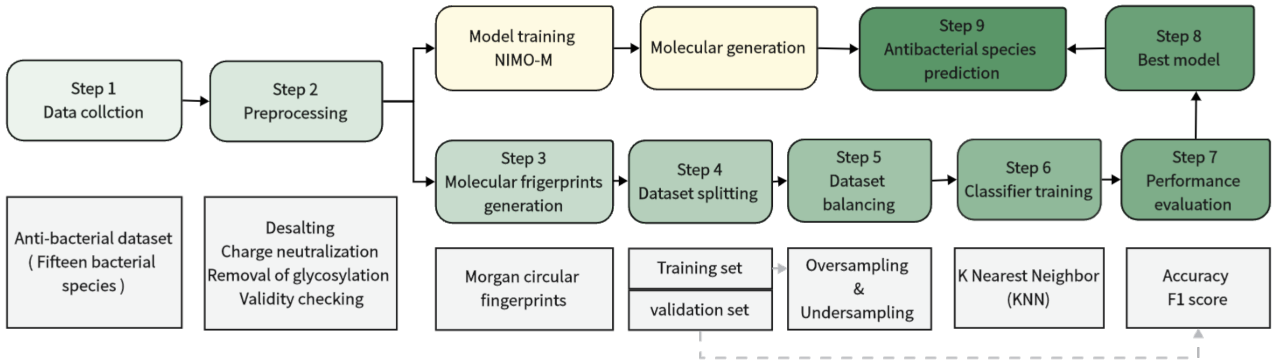

Figure S8. Workflow of designing antibacterial discovery libraries.

6. Canonicalization of fragment sequence

As shown in Figure S9 and Figure S10, the fragment extraction results in initial fragments containing dummy atoms. The bond id in the original molecular G is retained as map id to mark the location where two fragments can join together, e.g. [2*]=C([8*])O, with map id 2 and 8. The optional step in Method 2 tries to divide the rings of the scaffold into two equal parts as much as possible. Ring separation would result in two-part fragments containing a common bond and the carbon atom on the common bond will be replaced by the dummy atom [*]. The initial fragments are canonicalized to form the final motif sequence. The procedure consists of the following steps.

Step 1: Check if the first map id of the first and second fragments from the initial sequence is the same. If the same, turn to Step 3. If different, turn to Step 2.

Step 2: Find whether the sequence has a fragment with the same first map id of the first fragment, if found, swap to the second position and turn to Step 3, otherwise change the initial sequence order and start from Step 1.

Step 3: Merge the first site of two fragments to generate an intermediate molecule. This intermediate is cyclically executed with the next fragment referring to Step 1. If all the fragments are consumed, turn to Step 4.

Step 4: Record the sequence of fragment merging and remove map id to form the final motif sequence.

Assume we have a five-initial fragment list generated by the above fragmentation algorithm. The canonicalization of fragment sequence consists of the following changes as shown in Table S2. The final semantic motif sequence allows us to reconstruct the molecule based on the later molecular reconstruction rules that no longer rely on dummy atom IDs.

Table S2. An example of canonicalization of fragment sequence

**Initial fragments**	[2*]C1CC(=O)NC(=O)C1, [10*][C@H]1CC([13*])CC([16*])C1=O, [16*]O, [13*]O, [2*]CC([10*])C
First merge	[2*]C1CC(=O)NC(=O)C1, [10*][C@H]1CC([13*])CC([16*])C1=O, [16*]O, [13*]O, [2*]CC([10*])C
Second merge	[10*][C@H]1CC([13*])CC([16*])C1=O, [10*][C@H]1CC([13*])CC([16*])C1=O, [16*]O, [13*]O
Third merge	[13*]C1CC([16*])C(=O)[C@@H](C(C)CC2CC(=O)NC(=O)C2)C1, [16*]O,[13*]O
Last merge	[16*]C1CC(O)C[C@H](C(C)CC2CC(=O)NC(=O)C2)C1=O, [16*]O
**Reconstructed molecule**	CC(CC1CC(=O)NC(=O)C1)[C@H]1CC(O)CC(O)C1=O
**The sorted fragments list**	[2*]C1CC(=O)NC(=O)C1, [2*]CC([10*])C, [10*][C@H]1CC([13*])CC([16*])C1=O, [13*]O, [16*]O
**The final motif sequence**	[*]C1CC(=O)NC(=O)C1, [*]CC([*])C, [*][C@H]1CC([*])CC([*])C1=O, [*]O, [*]O

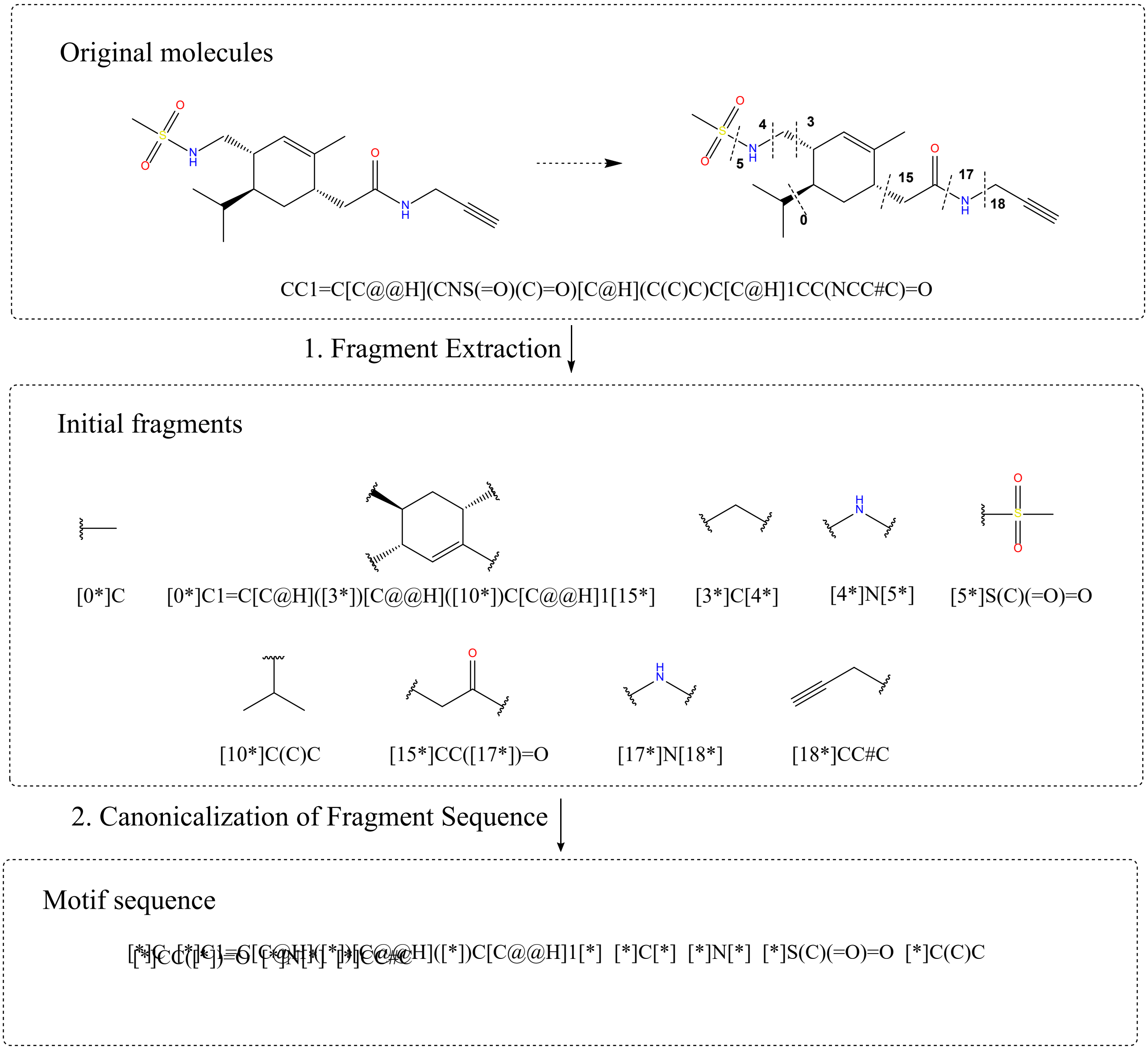

Figure S9. Overview of motif sequence generation for NIMO-M.

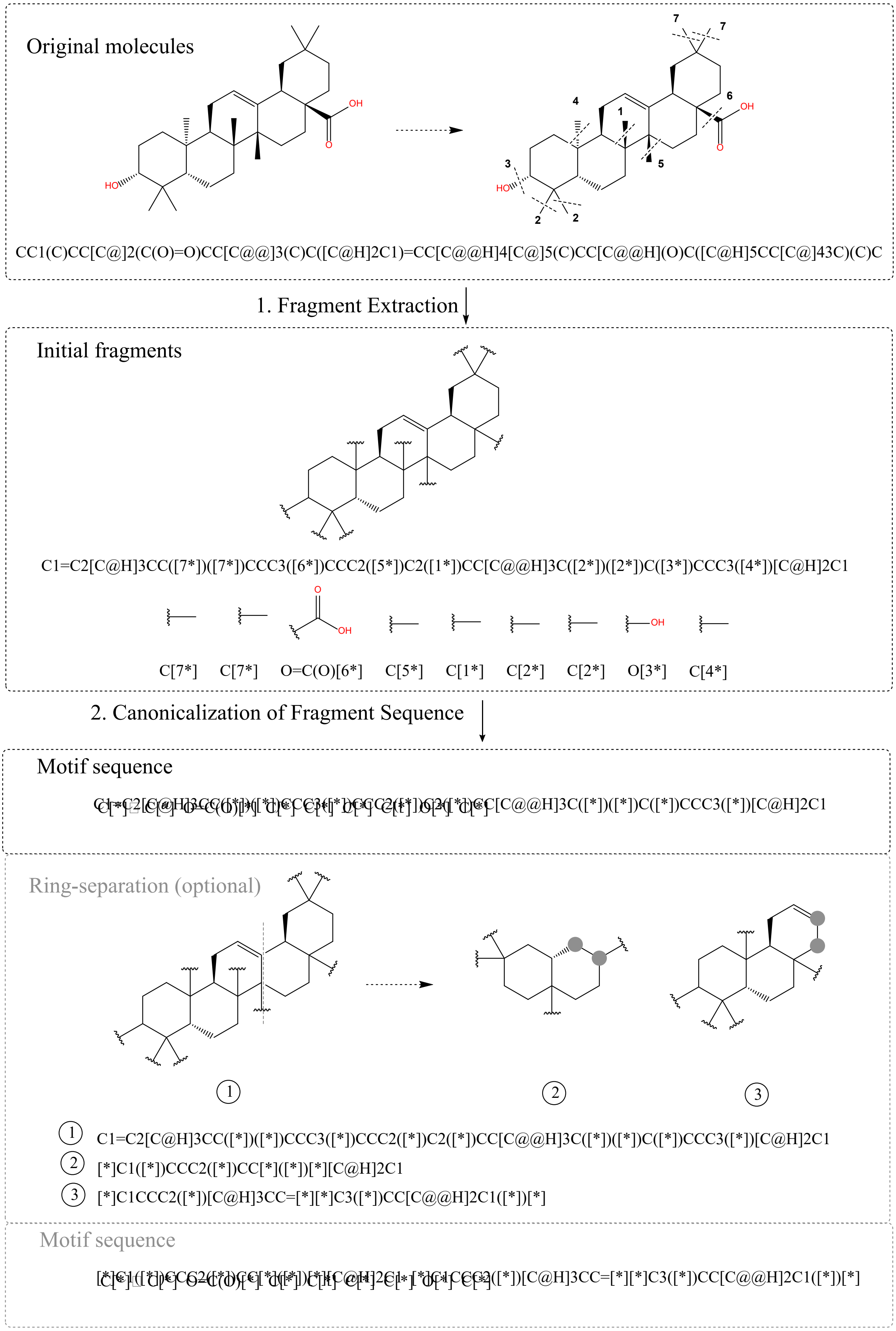

Figure S10. Overview of motif sequence generation for NIMO-S.

7. Molecular reconstruction verification

We examined each sequence based on the verification algorithm. The motif sequence which failed the validation step would be filtered out and not fed into the model. As illustrated in Figure S11, the molecular reconstruction verification did not consider the chiral difference, which meant that the recombinant molecular structure after elimination of chirality was the same as the original molecular structure, that is, it was marked as a successful reconstruction.

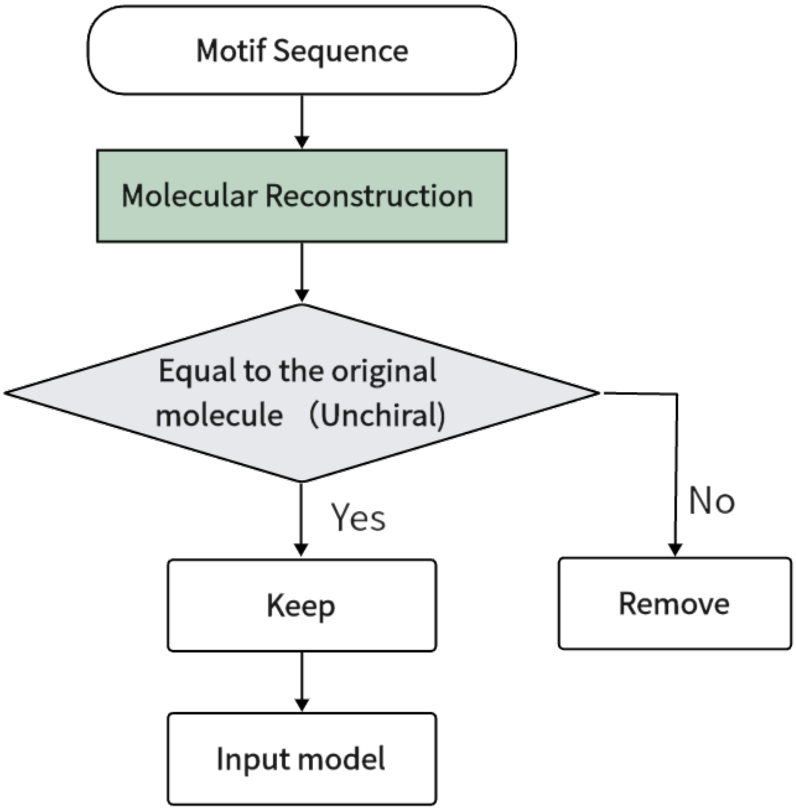

Figure S11. The decision flow chart of molecular reconstruction verification.

Figure S12 briefly describes an example of molecular reconstruction. The motifs are joined sequentially from left to right. In each step, the first dummy atoms in the form of SMILES are linked to a new chemical bond. The process works until all motifs have been consumed and there are no excess dummy atoms in the final product. Failed reconstructions mainly include the following cases: 1) the molecular reconstruction ends prematurely due to the intermediate not containing dummy atoms; 2) All motifs have been consumed and the final product still contains excess dummy atoms; 3) Bonds with dummy atom attached retain the information of the original bonds (single, double). If two different types of bonds are encountered, they cannot be joined according to the rules, and this motif sequence cannot successfully compose a molecule. Cases of failed reconstruction will reduce the validity of the generated molecules.

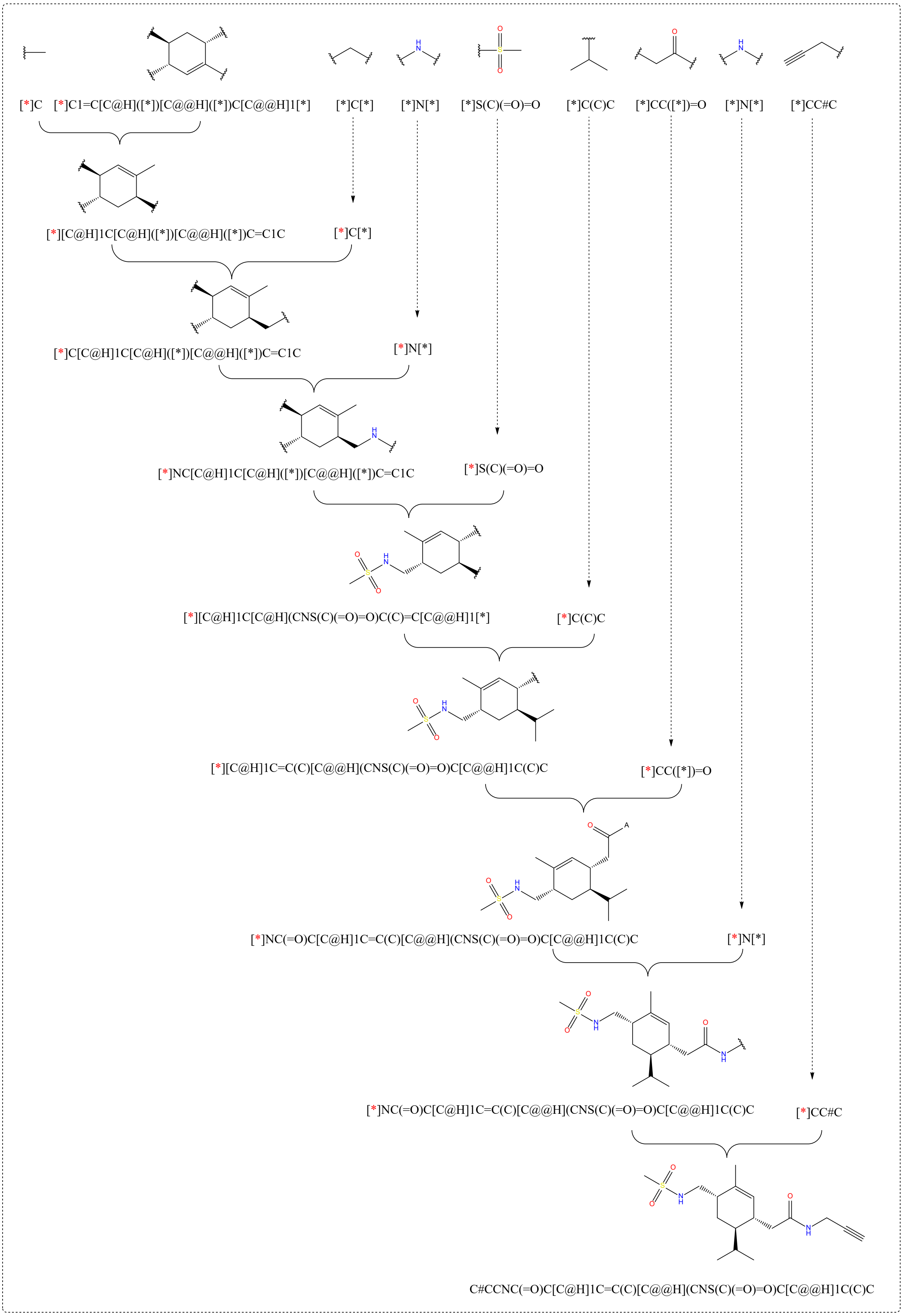

Figure S12. Overview of molecular reconstruction.

8. Model training and optimization of hyperparameters

8.1. Impact of *Beam width*

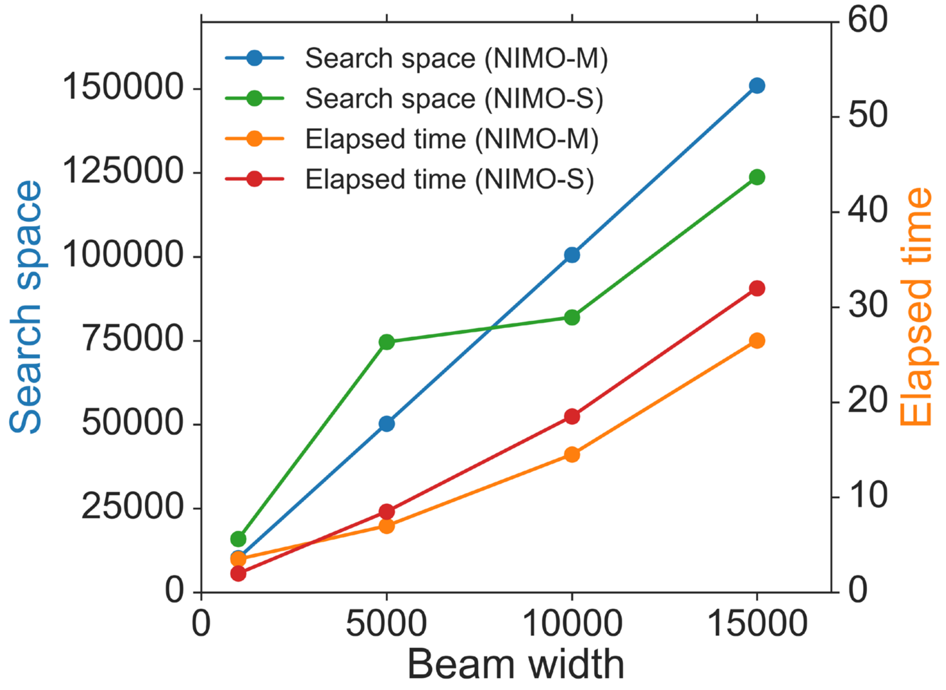

Figure S13. Search space and elapsed time (in minutes) of NIMO with varying settings of beam width.

Beam width is a hyperparameter in the adopting beam search algorithm. *Beam width* affects the maximum search space in one sampling process. As shown in Figure S13, the maximum search space and elapsed times showed a linear growth trend based on *Beam width*. Take NIMO-M as an example, when *Beam width* = 15,000, our model can provide up to 151,001 candidates in one sampling procedure with an elapsed time of 26 minutes.

8.2. Optimization of hyperparameters

NIMO was implemented on OpenNMT[6]. All scripts were written in Python (version 3.7). We trained the models on GPU (Tesla V100). Model automatically logs the training and validation process and visualizes the data using tensorboard tool. During the training process, the model state is saved as a checkpoint per 2,500 steps. The best hyperparameters were obtained based on the metrics including cross-entropy loss (ACE) and average accuracy (ACC). During continuous iterations, the ACE of the training set decreases and converges slowly, while the ACE of the validation set increases, and the ACC appears to subsequently increase and stabilize. Meanwhile, the dropout layers and early stopping steps are set to prevent the model from overfitting.

Hyperparameters mainly include the number of decoder layers, learning rates, batch size, and dropout rate. Batching examples with similar lengths together can minimize padding and facilitate the use of computing memory. Meanwhile, in batching scheme, the dynamic dataset iterator enables each training step can accept data of different batch sizes for iteration. We finally select our model with the best hyperparameters list in Table S3.

Table S3. Some key hyperparameters in NIMO

**Hyperparameters**	**Value**
Batch size	2,048
Train steps	50,000
Hidden states	512
Decoder layers	6
Attention heads	8
Optimizer	Adam
Dropout	0.1
Lr scheduler	Noam_decay
Starting learning rate	1.4
Warmup steps	8,000

9. References

1. Polykovskiy, D.; Zhebrak, A.; Sanchez-Lengeling, B.; Golovanov, S.; Tatanov, O.; Belyaev, S.; Kurbanov, R.; Artamonov, A.; Aladinskiy, V.; Veselov, M.; Kadurin, A.; Johansson, S.; Chen, H.; Nikolenko, S.; Aspuru-Guzik, A.; Zhavoronkov, A., Molecular Sets (MOSES): A Benchmarking Platform for Molecular Generation Models. *Front Pharmacol* **2020,** 11, 565644.
2. Sorokina, M.; Merseburger, P.; Rajan, K.; Yirik, M. A.; Steinbeck, C., COCONUT online: Collection of Open Natural Products database. *J Cheminform* **2021,** 13, (1), 2.
3. Landrum, G., RDKit: A software suite for cheminformatics, computational chemistry, and predictive modeling. *Greg Landrum* **2013**.
4. Bosc, N.; Felix, E.; Arcila, R.; Mendez, D.; Saunders, M. R.; Green, D. V. S.; Ochoada, J.; Shelat, A. A.; Martin, E. J.; Iyer, P.; Engkvist, O.; Verras, A.; Duffy, J.; Burrows, J.; Gardner, J. M. F.; Leach, A. R., MAIP: a web service for predicting blood-stage malaria inhibitors. *J Cheminform* **2021,** 13, (1), 13.
5. Wang, J.; Hsieh, C.-Y.; Wang, M.; Wang, X.; Wu, Z.; Jiang, D.; Liao, B.; Zhang, X.; Yang, B.; He, Q.; Cao, D.; Chen, X.; Hou, T., Multi-constraint molecular generation based on conditional transformer, knowledge distillation and reinforcement learning. *Nat*

Mach Intell **2021,** 3, (10), 914-922.
6. Klein, G.; Kim, Y.; Deng, Y.; Senellart, J.; Rush, A. M., Opennmt: Open-source toolkit for neural machine translation. *arXiv preprint arXiv:1701.02810* **2017**.
